# Effects of nitrogen addition on species composition and diversity of early spring herbs in a Korean pine plantation

**DOI:** 10.1002/ece3.10498

**Published:** 2023-09-05

**Authors:** Guanghui Yang, Mengmeng Zhang, Guangze Jin

**Affiliations:** ^1^ Center for Ecological Research Northeast Forestry University Harbin China; ^2^ College of Life Science Heilongjiang University Harbin China; ^3^ Key Laboratory of Sustainable Forest Ecosystem Management‐Ministry of Education Northeast Forestry University Harbin China; ^4^ Northeast Asia Biodiversity Research Center Northeast Forestry University Harbin China

**Keywords:** early spring herbs, Korean pine plantation, nitrogen addition, soil property, species diversity, understory light

## Abstract

Under the background of global nitrogen deposition, temperate forest ecosystems are suffering increasing threats, and species diversity is gradually decreasing. In this study, nitrogen addition experiments were conducted on Korean pine (*Pinus koraiensis*) plantations in Northeast China to explore the effect of long‐term nitrogen addition on herb species diversity to test the following hypothesis: long‐term nitrogen addition further reduced plant species diversity by affecting plant growth, which may be due to soil acidification caused by excessive nitrogen addition. Experimental nitrogen addition was conducted from 2014 to 2021, and the nitrogen treatment levels were as follows: N0 (control treatment, 0/(kg N ha^−1^ year^−1^)), N20 (low nitrogen treatment, 20/(kg N ha^−1^ year^−1^)), N40 (medium nitrogen treatment, 40/(kg N ha^−1^ year^−1^)) and N80 (high nitrogen treatment, 80/(kg N ha^−1^ year^−1^)). A herb community survey was conducted in the region from 2015 to 2021. The results showed that long‐term nitrogen addition decreased soil pH, changed the species and composition of herbaceous plants, and decreased the species diversity of understory herbaceous plants. With the increase in nitrogen application years, middle‐ and high‐nitrogen treatments significantly reduced the diversity of early‐spring flowering herbs and early‐spring foliating herbs, and their diversity decreased with the decrease in soil pH, indicating that soil acidification caused by long‐term nitrogen addition may lead to the decrease of plant diversity. However, for early‐spring growing herbs, adequate nitrogen addition may promote their growth. Our results show that plants have evolved different life‐history strategies based on their adaptation mechanisms to the environment, and different life‐history strategies have different responses to long‐term nitrogen addition. However, for most plants, long‐term nitrogen application will have a negative impact on the growth and diversity of herbs in temperate forests.

## Graphical abstract

Long‐term nitrogen addition reduced early spring herb species. Long‐term nitrogen addition significantly reduced the diversity of early‐spring flowering herbs and early‐spring foliating herbs. Soil acidification might be the main mechanism of species diversity reduction.

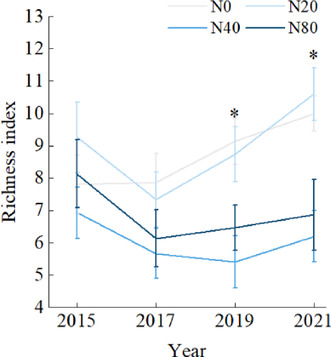

## 1 INTRODUCTION

Over the past decades, with the acceleration of industrial, agricultural and livestock processes, the global growth rate of human‐generated reactive nitrogen has accelerated dramatically (Galloway & Cowling, 2002; Gruber & Galloway, 2008). From 1980 to 2010, China increased 0.41 kg of nitrogen per hectare (kg N ha^−1^) on average annually. These reactive nitrogen emissions accelerated the atmospheric nitrogen deposition rate, greatly affecting plant growth, biodiversity, ecosystem carbon exchange and nitrogen transformation processes and threatening the ecosystem health of the entire northern hemisphere temperate zone and northern forests (Liu et al., 2011). Therefore, exploring the impact of nitrogen deposition on biodiversity is extremely important for understanding the impact of human activities on biodiversity (Roth et al., 2015).

In recent years, many studies have focused on the impact of nitrogen deposition on herbaceous species (Stevens et al., 2011; Wu et al., 2013). Some studies have found that the loss rate of species diversity in areas with long‐term nitrogen deposition is higher, and some species may even become extinct (Clark & Tilman, 2008; Lin et al., 2021; Stevens et al., 2004). In addition, The quantitative characteristics (including coverage and density) of herbaceous plants also had a negative response to nitrogen addition (Lai et al., 2018). There are many reasons for these effects. The nitrogen homogeneity hypothesis (Gilliam, 2006), colonization limitation hypothesis (Tilman, 1993), secondary stress theory (Moffat, 1998) and light competition theory (Hautier et al., 2009) indicate that long‐term nitrogen addition will affect plant colonization, growth and survival. However, previous studies have shown that the growth and diversity of herbaceous plants in temperate regions are affected by long‐term nitrogen addition causes more significant soil acidification (Lai et al., 2018; Van Den Berg et al., 2011), the soil acidification hypothesis suggests that long‐term nitrogen addition causes soil acidification, reduces base cation content in soil (Magill et al., 2004), affects nutrient availability, thereby affecting the growth of aboveground and underground parts of plants (Li et al., 2015), and reduces plant diversity (Lu et al., 2010; Zhang & Han, 2012). Therefore, it is of great significance to understand the response mechanism of herbaceous plants to nitrogen deposition.

A life‐history strategy is the optimal allocation of resources to maintain plant growth and reproduction, which can maximize the fitness of species (Ban, 1995). In the mutual adaptation of plants to the environment, different plant traits and strategies will evolve (Taylor et al., 1990), forming a specific life‐history strategy of species. Early spring herbaceous plants are the early stage of the dynamic change of herbaceous layer in forest ecosystem and the special layer structure of forest ecosystem. According to the differences in life‐history strategies, the species can be divided into three categories: (1) early‐spring growing herbs: plants complete life cycle stages such as growth, flowering and fruit in early spring and fall dormancy after stand closure; (2) early‐spring flowering herbs: the life cycle stages of growth, flowering and fruiting are carried out in early spring and can continue to grow after stand closure; and (3) early‐spring foliating herbs: the plants that completed the growth process, flowering and fruiting after the stand closed (Wu et al., 1999). In the same period, plants with different life‐history strategies are in different growth and development stages, and plants in different life‐history stages have different responses to nitrogen deposition (Meyer‐Grünefeldt et al., 2015). Studies have shown that nitrogen deposition can affect plant growth, population dynamics, seed quality and reproduction by affecting the absorption of plant roots, leaves and inflorescences (De Frenne et al., 2018), thus further affecting plant species composition and diversity. Previous studies generally focused on exploring the effect of nitrogen addition on herb diversity during the most vigorous growth period of herbaceous plants (Bai et al., 2010; Gilliam, 2019; He et al., 2021), but there are few studies on specific herb plant communities in early spring (Phillips et al., 2021). Because early spring herbaceous plants can absorb and accumulate a large amount of nitrogen and potassium in the soil, they alleviate the loss of soil nutrients caused by early spring snowmelt and serve as a short‐term nutrient sink to store nutrients for forest ecosystems (Muller & Bormann, 1976). Moreover, the characteristics of plant decomposition promote the material cycle of forests and play an important ecological role in ecosystems (Wu et al., 1999). Therefore, under the background of global nitrogen deposition, exploring the mechanism of experimental nitrogen addition on its growth and diversity is of great significance for the protection, development and utilization of early spring herb resources.

Mixed broadleaved‐Korean pine (*Pinus koraiensis*) forest is the top zonal vegetation in Northeast China. Due to a large amount of industrial deforestation, the area of Korean pine forest decreased sharply in the early 20th century. Natural Korean pine was gradually replaced by natural secondary forest and artificial forest. The Korean pine plantation has become one of the main artificial forests in Northeast China and is distributed in three provinces of Northeast China. To explore the response mechanism of early spring herbs to nitrogen addition, we proposed the following hypothesis: long‐term nitrogen deposition affects plant growth and reduces species diversity by reducing soil pH. To test the above hypothesis, this study conducted long‐term experimental nitrogen addition in a Korean pine plantation in the Xiaoxing'an Mountains to explore the effects of long‐term simulated nitrogen addition experiment on early spring herbs, including: (1) the response of soil properties and the understory light environment to nitrogen addition; (2) the effects of the 7‐year nitrogen addition experiment on the species composition and quantitative characteristics of early spring herbs; and (3) the effects of long‐term nitrogen addition on the diversity of understory herbaceous plants and different life‐history strategies in early spring.

## 2 MATERIALS AND METHODS

### 2.1 Study site

The study area is the Heilongjiang Liangshui National Nature Reserve in Daqingshan County, Yichun City, Heilongjiang Province, China (47°10′50′′ N, 128°53′20′′ E). The reserve has rich forest resources and a complex community structure. *Pinus koraiensis* is the constructive species, mixed with *Betula costata*, *Tilia amurensis*, *Tilia mandshurica*, *Ulmus davidiana* var. *japonica*, *Fraxinus mandshurica*, *Phellodendron amurense*, *Juglans mandshurica* and several kinds of Acer sp. The vegetation coverage is 96%. It is hot in summer, and abundant in precipitation, which is generally concentrated in June–August. It is dry and cold in winter. The annual average temperature is −0.3°C, the annual average precipitation is 676 mm, the annual average relative humidity is 78%, and the snow period is 130–150 days. The landform of the protected area is a low hilly area with an elevation of 280–707 m and an average altitude of approximately 400 m. Dark brown soil is its zonal soil, accounting for 84.91% of the total protected area.

### 2.2 Experimental treatments

**FIGURE 1:**
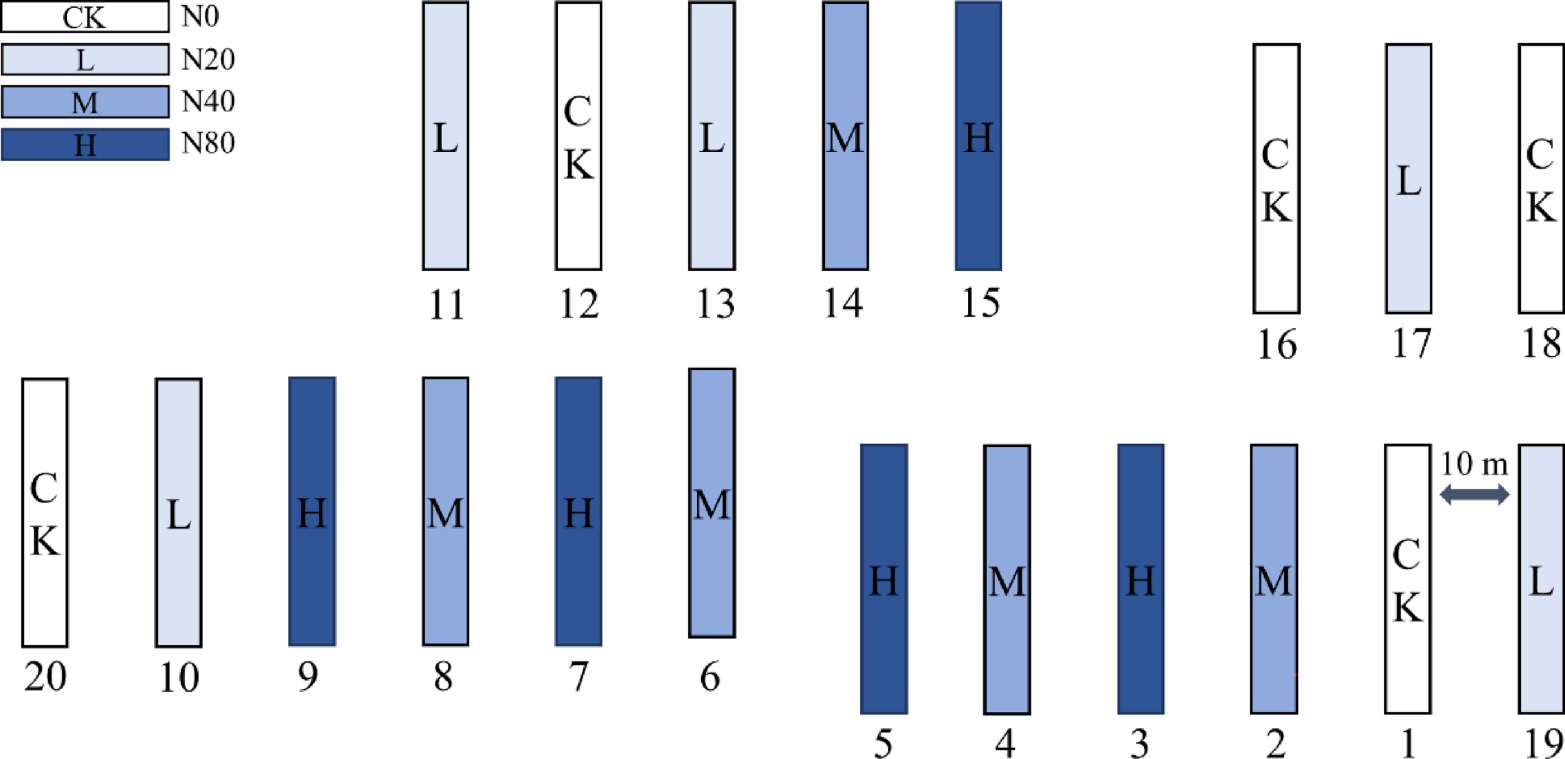
Schematic diagram of simulated N deposition experiment.

Nitrogen addition experiments were conducted under Korean pine plantation from 2014 to 2021, with the same amount of nitrogen applied from mid‐June to mid‐September. Twenty quadrats with basically identical site conditions of 5 m × 30 m were established in the forest, and each quadrat was separated by a 10 m interval band to eliminate the interference between the quadrats (Figure 1). The randomized block design was carried out in the sample plot, with 20/(kg N ha^−1^ year^−1^) as the standard of low nitrogen treatment, and the coefficient increased by 2 times. Four nitrogen concentration gradients (5 replicates) were set: N0 (control treatment, 0/(kg N ha^−1^ year^−1^)), N20 (low nitrogen treatment, 20/(kg N ha^−1^ year^−1^)), N40 (medium nitrogen treatment, 40/(kg N ha^−1^ year^−1^)) and N80 (high nitrogen treatment, 80/(kg N ha^−1^ year^−1^)). The nitrogen source was ammonium nitrate (NH_4_NO_3_), which was dissolved in 20 L water, and the solution was evenly sprayed on the soil surface of the nitrogen application area by a backpack sprayer. To avoid the difference between samples caused by water application, the same amount of deionized water was applied in the control group (Yang et al., 2019).

### 2.3 Field sampling

Early spring herb diversity surveys were conducted in mid‐May 2015–2021 (Appendix S1). Three 1 m × 1 m quadrats were randomly set for each quadrat to investigate the species, individual number of species (counted by above‐ground individual plants), height and coverage of early spring herbs in the quadrat. The coverage was determined by the grid method. The 1 m × 1 m quadrat was divided into 100 small grids of 10 cm × 10 cm, and the coverage was estimated by the visual method. In the actual survey of the plot, it was found that some of the plots that had undergone nitrogen addition experiments had no herb growth, which may lead to missing data in some plots.

In mid‐May 2021, a digital camera (Coolpix 4500; Nikon) connected to a 180° fisheye lens (Nikon FC‐E8) was used to collect canopy hemispheric effects at the 1.3 m centre of each herb sample. Gap Light Analyzer 2.0 (GLA) image processing software was used to process forest canopy image photos (Frazer et al., 1999). Transmitted direct light (TDir), transmitted diffuse light (TDif) and transmitted total light (TTot) were calculated.

Soil samples at 0–5 cm (a) and 5–10 cm (b) layers in the quadrat were collected in 2021, and the samples were packaged in polyethylene bags and brought back to the laboratory for the determination of chemical properties. After natural drying, the soil was tested in the laboratory through 2 and 0.149 mm soil sieves. A carbon and nitrogen analyzer (multiN/C 3000, Germany) was used to determine the total carbon (TC) content by the burning method, and total nitrogen (TN) was determined by an AQ2 discrete analyzer (SEAL Analytical, Inc.). An acidimeter (HANNA pH 211, Ital) was used to determine the pH value of the soil samples (soil‐liquid ratio: 1:2.5).

### 2.4 Statistical analysis

The data of early spring herbs were sorted in Excel, and the community number characteristics of early spring herbs were calculated, including individual number, relative height (*H*_*i*_), relative density (*D*_*i*_), relative coverage (*C*_*i*_) and importance value (*P*_*i*_). The formula is as follows: importance value (Zhang et al., 2021):

$$P_{i}=\frac{\left(D_{i}+C_{i}+H_{i}\right)}{3}$$

The vegan package in R 4.0.2 software was used to calculate the species richness of herbaceous plant communities in early spring. The formula is as follows:

$$\text{Species richness}:S=N_{s}$$

Two‐way ANOVA and linear mixed model were performed on early spring herb data in 2015, 2017, 2019 and 2021 using R 4.0.2 software to study the overall effect of early spring plant diversity during N treatment over time. In order to compare the changes of height, coverage, and density of early spring herbaceous plants after 7 years of nitrogen application, the herbaceous data of 2015–2021 were used for one‐way analysis of variance (one‐way ANOVA), and the least significant difference (LSD) method with Bonferroni adjustment was used for analysis to explore the differences in nitrogen treatment levels of early spring herbs in three life‐history strategies, The same method was used to calculate the annual response of richness index under different treatments. The soil chemical properties were analyzed by one‐way ANOVA to compare the changes in soil properties between different treatments. LN conversion of the data. The hier.part (Nally & Walsh, 2004) package in R 4.0.2 was used to analyze the environmental factors and plant growth data in 2021, and the correlation analysis was used to explain the effects of environmental factors changes caused by nitrogen addition on the quantitative characteristics and diversity of herbaceous plants. All graphs were drawn in Origin 2019.

## 3 RESULTS

In 2015, a total of 38 species of plants were found under the forest, and in 2021, a total of 36 species of plants were investigated under the forest (Appendix S2). Nitrogen application for 7 years did not cause loss of early‐spring growing herbs and early‐spring flowering plant species, but reduced the species of early‐spring foliating herbs.

### 3.1 Responses of soil and light to experimental nitrogen addition under the Korean pine plantation

**FIGURE 2:**
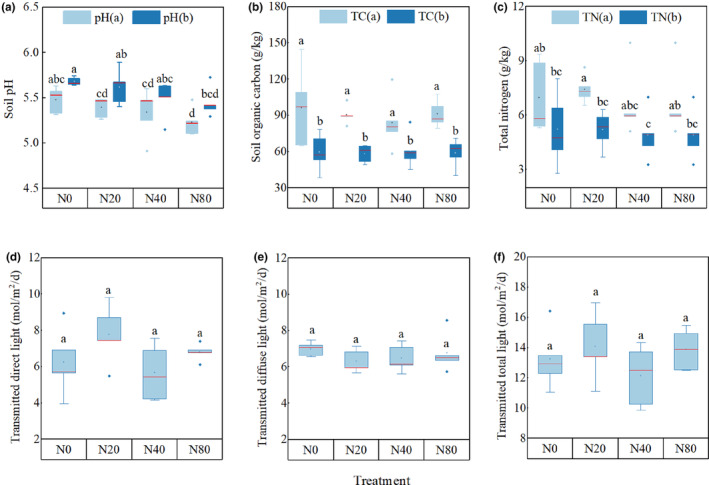
Descriptive statistics of soil and light in the Korean pine plantation under nitrogen addition. Values are the means ± SE. pH(a): 0–5 cm soil pH; pH(b): 5–10 cm soil pH; TC(a): 0–5 cm total carbon; TC(b): 5–10 cm total carbon; TN(a): 0–5 cm soil total nitrogen; TN(b): 5–10 cm soil total nitrogen; values are the means ± SE. Values with different letters are significantly different (*p* < .05).

Soil chemical properties of the Korean pine plantation were different after 7 years nitrogen addition experiment. The pH(a) of N20 was significantly lower than that of pH(b), and the pH(a) and pH(b) of N80 were significantly lower than those of N0 (Figure 2a), indicating that 7‐year nitrogen addition significantly reduced soil pH in the 0–5 and 5–10 cm soil layers, and soil TC and TN in the 0–5 cm layer were significantly higher than those in the 5–10 cm layer (Figure 2b,c). Light intensity under forest did not change significantly after 7 years of the experiment (Figure 2d–f).

### 3.2 Interannual response of herb species richness to nitrogen addition in early spring

**FIGURE 3:**
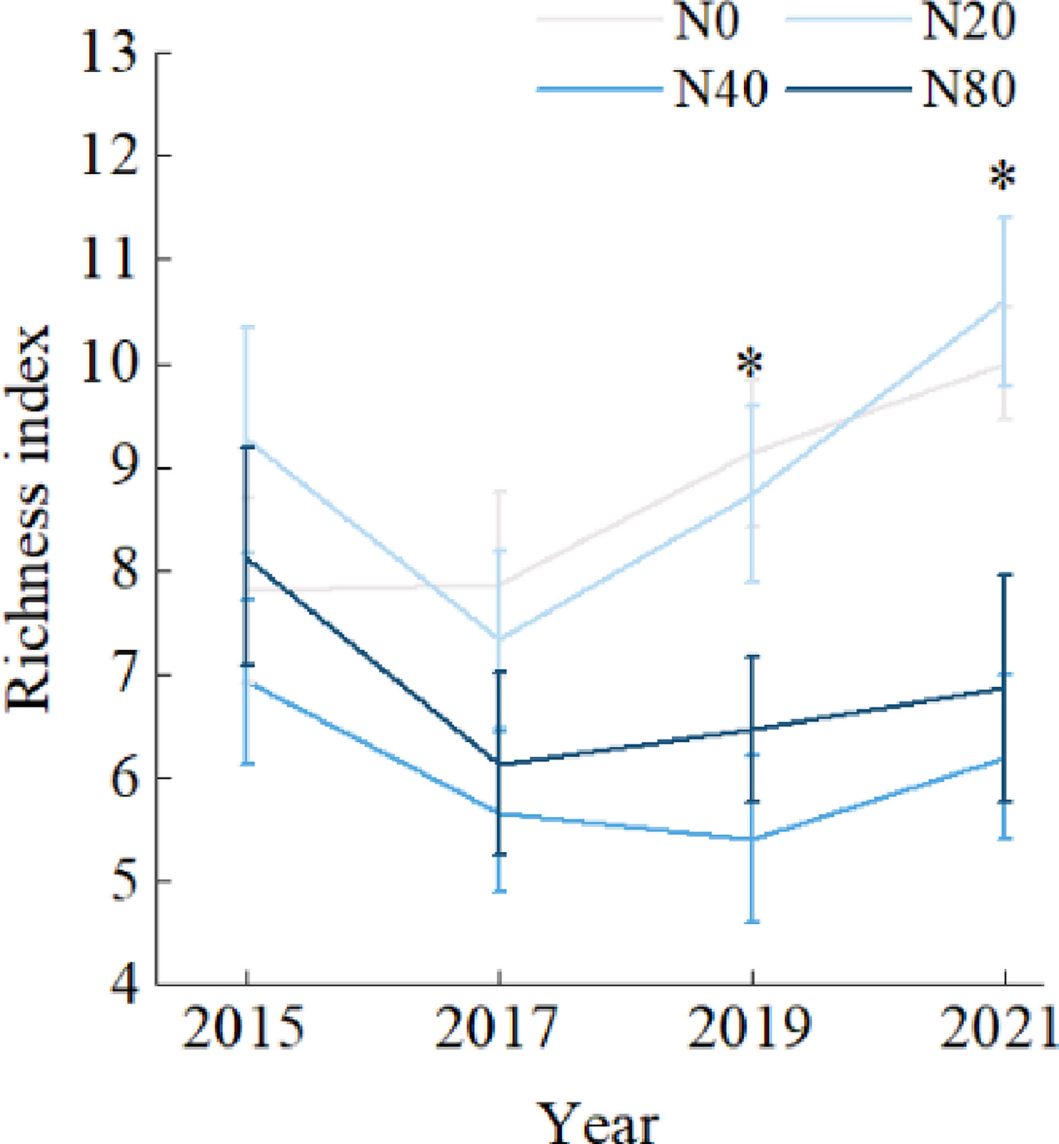
Interannual response of herb species richness to nitrogen addition in early spring. Values are the means ± SE. *Significant difference with N treatment plots (*p* < .05).

The species richness of early spring herbaceous plants to nitrogen addition treatment did not change significantly in 2015 and 2017, but in 2019 and 2021, medium nitrogen treatment and high‐nitrogen treatment significantly reduced the richness of early spring herbaceous plants (*p* < .05). With the increase in nitrogen application years, the species richness of early spring herbs in N0 and N20 showed an increasing trend. Compared with 2015, the species richness of N40 and N80 plots in 2021 decreased (Figure 3).

Nitrogen addition experiment had a significant effect on the richness of early spring herbaceous plants with three life‐history strategies (*p* < .05). The species richness of early‐spring flowering herbs and early‐spring foliating herbs changed with the increase of nitrogen application years (*p* < .05), The interaction between nitrogen application years and nitrogen concentration had no significant effect on the richness of herbaceous plants in different life‐history strategies (Table 1). However, the results of the linear mixed model showed that the effect of year change on the species richness of the three life‐history strategies was more significant (Appendix S4).

**TABLE 1:** Response of species richness of early spring‐herbaceous plants under different life‐history strategies to nitrogen rates and years in 2015, 2017, 2019 and 2021.

Effect	df	Life‐history strategy		
		Early‐spring growing herbs	Early‐spring flowering herbs	Early‐spring foliating herbs
Year	3	0.364 (0.779)	29.198 (**<0.001**)	7.201 (**<0.001**)
Nitrogen	3	4.175 (**0.007**)	20.345 (**<0.001**)	8.937 (**<0.001**)
Nitrogen × Year	9	0.778 (0.627)	1.461 (0.164)	0.391 (0.845)

*Note*: The *F*‐values and *p*‐values in parentheses are shown, bold values denote significant effects (*p* < .05).

**FIGURE 4:**
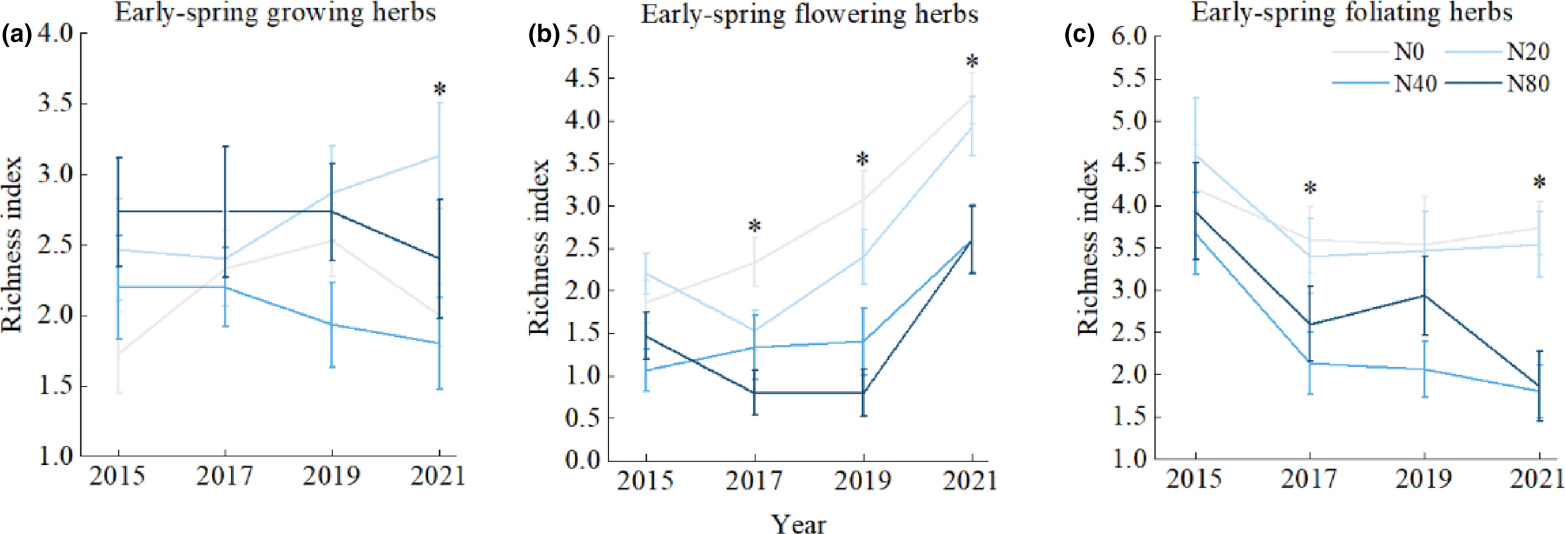
Response of early spring herb richness to nitrogen addition treatment and interannual variation under different life‐history strategies. Values are the means ± SE. *Significant difference with N treatment plots (*p* < .05).

In 2021, the species richness of early‐spring growing herbs under N20 treatment was significantly higher than that under N0 and N40 (Figure 4a). In 2017, 2019 and 2021, the species richness of early‐spring flowering herbs under N0 treatment was significantly higher than that under N40 and N80 (Figure 4b). In 2017 and 2021 the species richness of early‐spring foliating showed a significant decrease in N40 (Figure 4c). Low levels of nitrogen addition significantly increased the species diversity of early‐spring growing herbs, while medium and high levels of nitrogen addition significantly reduced the species diversity of early‐spring flowering herbs and early‐spring foliating herbs.

There are differences in the interannual variation of nitrogen application in plants with different life‐history strategies. Compared with 2015, the species richness of early‐spring growing herbs did not change significantly (Figure 4a), in 2021, the species richness of early‐spring flowering herbs under four nitrogen treatment levels showed an increasing trend (Figure 4b), and the species richness of early‐spring foliating herbs under high‐nitrogen treatment decreased gradually with the increase of nitrogen application year. (Figure 4c).

### 3.3 Effects of nitrogen addition on the composition and quantitative characteristics of early spring herbs with different life‐history strategies

**FIGURE 5:**
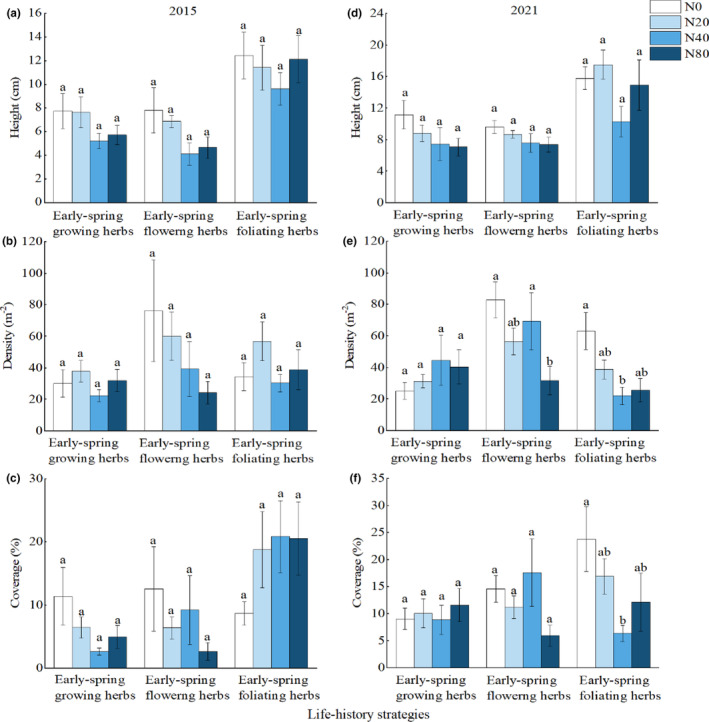
Effects of nitrogen addition on the quantitative characteristics of early spring herbs in different life‐history strategies. Values are the means ± SE. Values with different letters are significantly different (*p* < .05).

There were no significant differences in the height, density and coverage of early spring herbs among the three life‐history strategies at different nitrogen levels in the first year of nitrogen application (Figure 5a–c); in 2021, the density of early‐spring flowering herbs under the N80 treatment was significantly lower than that under the N0 and N40 treatments (Figure 5e), and the density and coverage of early‐spring foliating herbs under the N40 treatment were significantly lower than those under the N0 treatment (Figure 5e,f).

### 3.4 Relationship between quantitative characteristics and diversity of early spring herbs with different life‐history strategies and environmental factors

**FIGURE 6:**
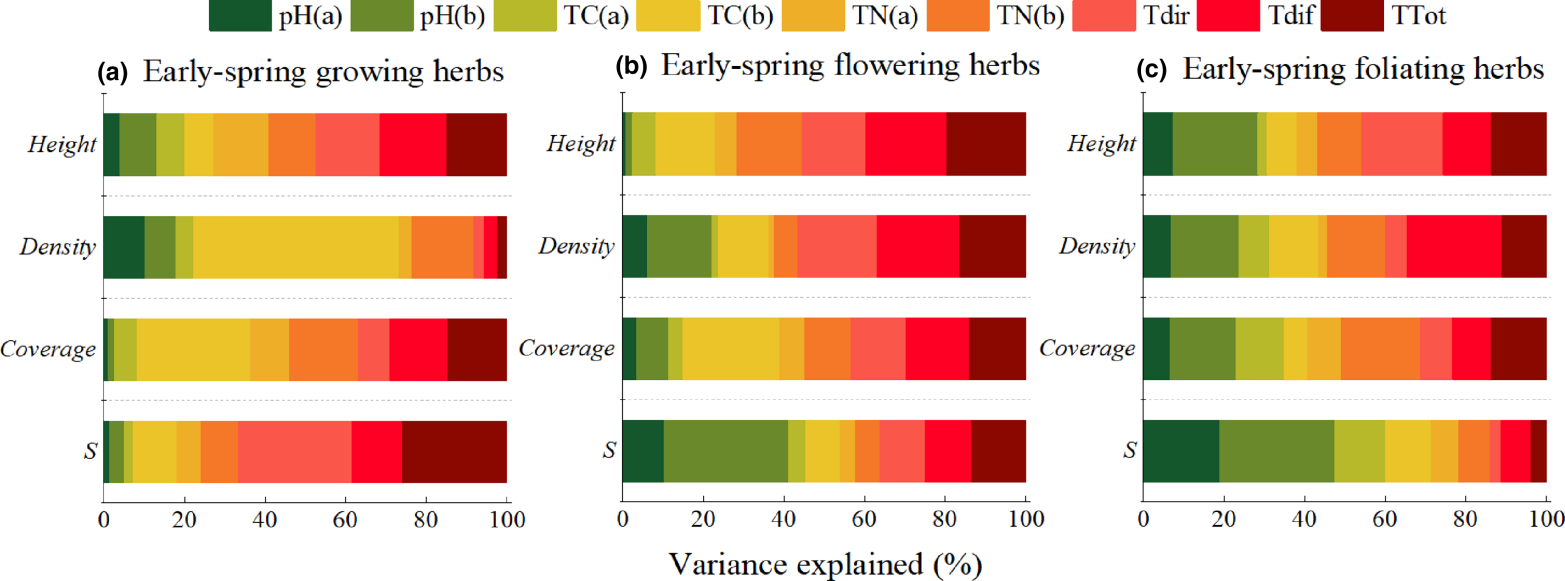
Hierarchical partitioning analysis results of environmental factors on quantitative characteristics and diversity index of early spring herbs. pH, soil pH; *S*, richness index; TC, total carbon; TDif, transmitted diffuse light; TDir, transmitted direct light; TN, soil total nitrogen; TTot, transmitted total light. The same below.

For early‐spring growing herbs, TC(b) and TN(b) had the highest explanatory rates for density and coverage, which were 28.0%, 17.1%, 50.9% and 15.5%, respectively, and the explanatory rates of understory light factors for the species richness index were >65% (Figure 6a). The transmitted diffused light and transmitted total light had the highest interpretation rates for the height and density of early‐spring flowering herbs. The interpretation rate of TC(b) for coverage was 24.0%. The pH(b) explained 30.8% of the richness index (Figure 6b). The transmitted diffused light had the highest interpretation rate (23.5%) for the density of early‐spring foliating plants, and the pH of the two layers of soil had a nearly 50% interpretation rate for the richness index (Figure 6c).

Environmental factors are closely related to the growth and diversity of herbaceous plants. The height and coverage of early‐spring growing herbs were positively correlated with TC(b), TN(a), TN(b), TDif and TTot, and their species richness index was positively correlated with TDir, TDif and TTot. For early‐spring flowering herbs, TC(b), TDif and TTot were significantly positively correlated with height and coverage, and the richness index was significantly positively correlated with pH(b) and TTot. The richness index of early‐spring foliating plants was significantly positively correlated with soil pH(a) and was extremely significantly positively correlated with soil pH(b) (Table 2).

**TABLE 2:** Correlation analysis of quantitative characteristics and diversity of early spring herbs in Korean pine plantation with environmental factors under nitrogen addition.

Life‐history strategies	Quantitative characteristics and diversity of species	Soil properties						Light factors		
		pH(a)	pH(b)	TC(a)	TC(b)	TN(a)	TN(b)	TDir	TDif	TTot
Early‐spring growing herbs	Height	0.051	0.295	0.452*	0.496*	0.544*	0.592**	0.500*	0.522*	0.549*
	Density	0.147	0.035	0.091	0.495*	0.135	0.263	0.000	0.210	0.110
	Coverage	0.049	0.105	0.361	0.646**	0.450*	0.590**	0.365	0.514*	0.511*
	Species richness	0.023	0.218	0.181	0.374	0.304	0.334	0.619**	0.461*	0.657**
Early‐spring flowering herbs	Height	−0.035	0.061	0.390	0.573**	0.382	0.615**	0.503*	0.584**	0.620**
	Density	0.235	0.381	0.111	0.293	0.127	0.222	0.285	0.333	0.297
	Coverage	0.203	0.336	0.299	0.529*	0.350	0.440	0.381	0.466*	0.460*
	Species richness	0.337	0.565**	0.318	0.382	0.279	0.306	0.415	0.474*	0.503*
Early‐spring foliating herbs	Height	0.079	0.207	0.064	0.020	0.139	0.166	0.217	−0.146	0.073
	Density	0.253	0.361	0.268	0.240	0.138	0.068	0.080	0.462*	0.258
	Coverage	0.274	0.391	0.328	0.086	0.221	−0.066	0.097	0.301	0.242
	Species richness	0.532*	0.609**	0.469*	0.401	0.350	0.257	0.172	0.398	0.281

*Note*: The bold values denote significantly correlated. *Significantly correlated between species and environment factors (*p*  <  .05), and **extremely significantly correlated between species and environment factors (*p* < .01).

## 4 DISCUSSION

### 4.1 Effects of experimental nitrogen addition on the composition of early spring herbs

The results showed that there were two fewer plant species in 2021 than in 2015, and there was no obvious species decrease (Appendix S2). This result was inconsistent with Clark and Tilman (2008) and Stevens et al. (2004). From the perspective of different life‐history strategies, the number of early‐spring growing herbs and early‐spring flowering herbs in 2012 increased by 1 and 3 respectively, while the number of early‐spring foliating herbs decreased by 6 (Appendix S2). Studies have shown that in temperate regions under long‐term nitrogen deposition, nutrient‐demanding species prefer ongoing eutrophication, while the frequency of other species with low nitrogen demand will gradually decrease (Hedwall & Brunet, 2016), which may be the reason for the increase of plant species with short life history and high nutrient demand in early spring, and the decrease of plant species with long life history and low nitrogen demand in early spring. With the increase in nitrogen application years and concentrations, *Athyrium spinulosum* and *Adiantum pedatum* gradually disappeared from the forest (Appendix S2), but *Athyrium brevifrons* occupied a dominant position under various nitrogen application concentrations (Appendix S3), which may be because ferns were sensitive to changes in environmental factors (Nervo et al., 2019), but the sensitivity of different ferns to nitrogen was different.

### 4.2 Effects of experimental nitrogen addition on the diversity and growth of early spring herbs

In the forest ecosystem, experimental nitrogen addition had a significant effect on the richness index of understory herbaceous plants. Nitrogen addition significantly reduced species diversity (Figure 3), which was consistent with Stevens et al. (2010) and Li et al. (2020). Additionally, the input of experimental nitrogen changed the external environment of plant survival, and the responses of plants with three life‐history strategies to different nitrogen application concentrations were different. The results showed that the species richness of early‐spring growing herbs period in 2021 did not change significantly under the condition of middle‐ and high‐concentration of nitrogen addition, but increased significantly under low nitrogen concentration (Figure 4a), indicating that long‐term high concentration of nitrogen addition did not show significant inhibition on the growth of early‐spring growing herbs. In this period, early‐spring growing herbs, growing, flowering and fruiting, took advantage of the characteristics of a short life cycle and absorbed a large amount of N, K and other elements as a short‐term nutrient sink of forests in their survival process, nutrients are provided for the growth of forest plants (Muller & Bormann, 1976). Therefore, it will not be affected by nitrogen enrichment in soil, and even under appropriate nitrogen addition treatment, species diversity will increase slightly. The long‐term nitrogen addition experiment affected the density, coverage and diversity of early‐spring flowering herbs and early‐spring foliating herbs, and the species richness index in the middle‐ and high‐nitrogen areas showed significant decreases (Figure 4b,c), indicating that early‐spring flowering herbs and early‐spring foliating herbs were nitrogen‐sensitive plants and that excessive nitrogen addition significantly affected their growth and distribution. With the increase in nitrogen application years, the richness of early spring herbs in the medium‐ and high‐nitrogen application areas did not change significantly (Figure 3). However, in 2021, the richness of early‐spring flowering herbs showed an increasing trend (Figure 4b), while the richness of early‐spring foliating herbs decreased significantly (Figure 4c), indicating that although they were both sensitive plants, the adaptability of early‐spring flowering herbs to nitrogen was stronger than that of early‐spring foliating herbs at the same nitrogen application level. The response of plant growth and diversity to nitrogen addition is not consistent in the three life‐history strategies, which may be due to the different utilization of resources in different growth periods, so the restriction of resources on plants will be different. In addition, there are differences in the interannual variation of nitrogen application in plants with different life‐history strategies. Compared with 2015, the middle‐ and high‐nitrogen treatment after 7 years significantly increased the species richness of early‐spring flowering herbs and significantly reduced the species richness of early‐spring foliating herbs. The results showed that plant diversity changed with the increase in nitrogen application years, but plants had different responses to the interannual effects of long‐term nitrogen addition due to their own tolerance to nitrogen.

### 4.3 Responses of environmental factors to experimental nitrogen addition and effects on early spring herb diversity

Soil provides essential nutrients for plant growth and is the basis for plant growth and development (Nakamura et al., 2008). Long‐term nitrogen addition causes soil acidification and changes in other chemical properties (Magill et al., 2004). The results of this study confirmed that long‐term nitrogen addition significantly reduced soil pH (Figure 2). Studies have shown that long‐term nitrogen addition causes soil acidification, reducing the base cation content in soil (Magill et al., 2004), thereby affecting the growth of aboveground and underground parts of plants (Li et al., 2015) and reducing plant diversity. Since the soil in this region is acidic and the pH is generally between 4.8 and 6.0, the results of this study show that the diversity of early‐spring flowering herbs and early‐spring foliating herbs affected by experimental nitrogen addition is significantly positively correlated with soil pH (Table 2), and the species diversity was also reduced with the decrease of soil pH. Therefore, soil acidification may be one of the reasons for the decrease in herbaceous diversity in early spring. In addition, the height and coverage of early‐spring growing herbs and early‐spring flowering herbs were significantly positively correlated with soil organic carbon and total nitrogen because in the ecosystem, C and N are generally considered structural substances and nutrients for plant growth and development and play a positive role in promoting plant growth (Oldroyd & Leyser, 2020). Understory light is closely related to the growth of herbaceous plants, and the availability and heterogeneity of light have an important impact on the composition and richness index of the understory of herbaceous plants (Kumar et al., 2018). Resource competition theory shows that the addition of limiting elements in nitrogen‐limited stands will reduce the dimension of underground resource balance, resulting in a change in plant niche space from the common limitation of soil nutrients and light to the single limitation of light (Harpole et al., 2016), and the competition of plants for light is asymmetric. Higher plants can obtain more light than smaller plants, resulting in increased competitive exclusion between species (DeMalach et al., 2017) and the loss of plant species. However, in our study, the light factors were highly homogeneous at four nitrogen application concentrations (Figure 2), indicating that the light intensity available to understory plants was consistent, and the results showed that there was no significant difference in the heights of the three life‐history strategies at different nitrogen addition concentrations (Figure 5a,d), indicating that light competition was not the main mechanism for reducing the diversity of understory herbaceous plants.

## 5 CONCLUSION

Long‐term nitrogen addition significantly affected early spring herbaceous plants diversity and soil properties. The 7‐year nitrogen addition experiment reduced species diversity by reducing soil pH and inhibiting plant growth. Middle‐ and high‐nitrogen treatments significantly reduced the density, coverage and diversity index of early‐spring flowering herbs and early‐spring foliating herbs, while a small amount of nitrogen addition significantly increased the species richness of early‐spring growing herbs. Species with short life cycle and high nutritional demand can quickly absorb nutrients, promote their own growth, complete life activities and achieve a high level of species diversity under low nitrogen conditions, while long‐term high nitrogen addition by changing soil properties, reducing soil pH, destroying soil nutrient balance, thereby inhibiting plant growth and reducing species diversity. In addition, the diversity of early spring herbaceous plants changed with the increase in nitrogen application years, but there were different responses to the interannual effects of long‐term nitrogen addition due to the differences in plant tolerance to nitrogen. Soil acidification caused by long‐term nitrogen addition is one of the reasons for the decrease in species diversity. Ion toxicity and secondary stress of nitrogen may also cause the decrease of species diversity. On the basis of long‐term nitrogen addition treatment, more mechanisms affecting herbaceous plant diversity need to be further studied.

## AUTHOR CONTRIBUTIONS

**Guanghui Yang:** Conceptualization (equal); data curation (equal); formal analysis (equal); investigation (equal); methodology (equal); writing – original draft (equal). **Guangze Jin:** Conceptualization (equal); funding acquisition (equal); methodology (equal); project administration (equal); resources (equal); supervision (equal); writing – review and editing (equal). **Mengmeng Zhang:** Data curation (equal); writing – review and editing (equal).

## CONFLICT OF INTEREST STATEMENT

The authors declare that they have no known competing financial interests or personal relationships that could have appeared to influence the work reported in this paper.

## Supporting information


Appendix S1.
Appendix S2.
Appendix S3.
Appendix S4.

## Data Availability

Survey data of early spring herbs and code under Korean pine plantation are publically available at https://github.com/guangguang‐yang/data1.git.
