# Supplementary material for: Effects of nitrogen addition on species composition and diversity of early spring herbs in a Korean pine plantation
Source: Ecol Evol. 2023 Sep 5;13(9):e10498. doi: 10.1002/ece3.10498 (PMC10480043; doi:10.1002/ece3.10498)
Supplement: Supplementary file 1 — Appendix S1. Appendix S2. Appendix S3. Appendix S4. [file ECE3-13-e10498-s001.docx]

**Appendix S1** The schematic diagram of early spring herb quadrats under different nitrogen treatments

**
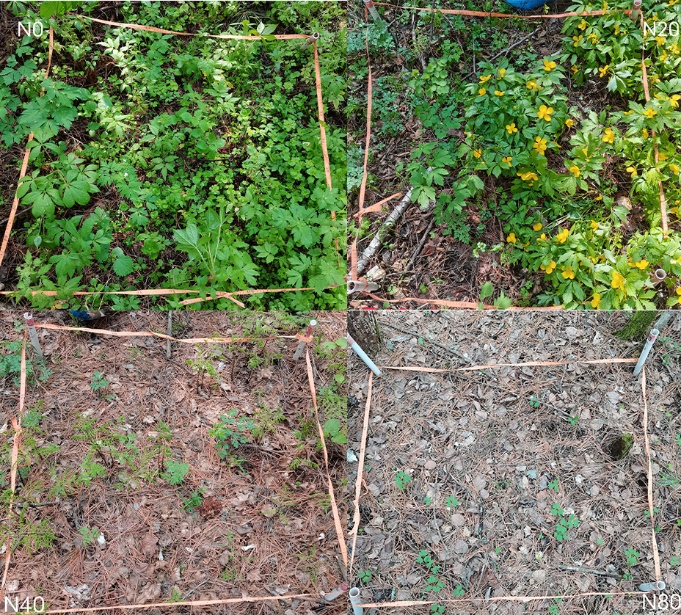
**

**Appendix S2** List of understory herbaceous plants in a Korean pine plantation with nitrogen addition in different years

| Life-history strategies | Species | 2015 | 2017 | 2019 | 2021 |
| --- | --- | --- | --- | --- | --- |
| Early-spring growing herbs | *Adonis amurensis* | — | — | √ | — |
| Early-spring growing herbs | *Adoxa moschatellina* | √ | √ | √ | √ |
| Early-spring growing herbs | *Anemone cathayensis* | √ | — | √ | √ |
| Early-spring growing herbs | *Corydalis yanhusuo* | √ | √ | √ | √ |
| Early-spring growing herbs | *Enemion raddeanum* | √ | √ | √ | √ |
| Early-spring growing herbs | *Eranthis stellata* | √ | √ | — | √ |
| Early-spring growing herbs | *Gagea nakaiana* | — | — | — | √ |
| Early-spring growing herbs | *Hylomecon japonica* | √ | √ | √ | √ |
|  |  |  |  |  |  |
| Early-spring flowering herbs | *Caltha palustris* | — | — | √ | — |
| Early-spring flowering herbs | *Cardamine leucantha* | — | √ | √ | √ |
| Early-spring flowering herbs | *Chelidonium majus* | — | — | √ | √ |
| Early-spring flowering herbs | *Chrysosplenium lectus-cochleae* | √ | √ | √ | √ |
| Early-spring flowering herbs | *Lamium barbatum* | √ | √ | √ | √ |
| Early-spring flowering herbs | *Moehringia lateriflora* | — | — | — | √ |
| Early-spring flowering herbs | *Oxalis corniculata* | √ | √ | √ | √ |
| Early-spring flowering herbs | *Paris verticillata* | √ | √ | — | √ |
| Early-spring flowering herbs | *Pseudostellaria davidii* | √ | √ | √ | √ |
| Early-spring flowering herbs | *Viola collina* | √ | √ | √ | √ |
|  |  |  |  |  |  |
| Early-spring foliating herbs | *Aconitum carmichaelii* | √ | √ | √ | √ |
| Early-spring foliating herbs | *Actaea asiatica* | √ | — | — | — |
| Early-spring foliating herbs | *Adiantum pedatum* | √ | — | √ | — |
| Early-spring foliating herbs | *Aegopodium alpestre* | √ | √ | √ | √ |
| Early-spring foliating herbs | *Aruncus sylvester* | √ | — | — | √ |
| Early-spring foliating herbs | *Athyrium multidentatum* | √ | √ | √ | √ |
| Early-spring foliating herbs | *Athyrium spinulosum* | √ | — | — | — |
| Early-spring foliating herbs | *Carex callitrichos* | √ | — | — | √ |
| Early-spring foliating herbs | *Carex pilosa* var. *auriculata* | √ | √ | √ | √ |
| Early-spring foliating herbs | *Carex siderosticta* | √ | — | — | — |
| Early-spring foliating herbs | *Circaea alpina* subsp. *caulescens* | — | — | √ | — |
| Early-spring foliating herbs | *Dryopteris crassirhizoma* | √ | √ | √ | √ |
| Early-spring foliating herbs | *Echinochloa crus-galli* | √ | — | — | — |
| Early-spring foliating herbs | *Equisetum hyemale* | √ | √ | √ | √ |
| Early-spring foliating herbs | *Filipendula palmata* | √ | √ | √ | √ |
| Early-spring foliating herbs | *Galium spurium* | √ | — | √ | √ |
| Early-spring foliating herbs | *Heracleum moellendorffii* | √ | — | — | — |
| Early-spring foliating herbs | *Impatiens noli-tangere* | √ | √ | √ | √ |
| Early-spring foliating herbs | *Maianthemum bifolium* | √ | √ | √ | √ |
| Early-spring foliating herbs | *Mitella nuda* | √ | √ | √ | √ |
| Early-spring foliating herbs | *Ophiopogon pierrei* | √ | √ | √ | √ |
| Early-spring foliating herbs | *Panicum miliaceum* | — | — | √ | √ |
| Early-spring foliating herbs | *Parasenecio auriculatus* | — | √ | — | — |
| Early-spring foliating herbs | *Parasenecio hastatus* | √ | — | — | √ |
| Early-spring foliating herbs | *Phryma leptostachya* subsp. *asiatica* | √ | — | — | √ |
| Early-spring foliating herbs | *Pilea pumila* | — | √ | — | — |
| Early-spring foliating herbs | *Rubia sylvatica* | √ | √ | √ | — |
| Early-spring foliating herbs | *Scutellaria baicalensis* | √ | — | — | — |
| Early-spring foliating herbs | *Thalictrum baicalense* | — | — | — | √ |
| Early-spring foliating herbs | *Urtica angustifolia* | √ | √ | — | √ |
| Early-spring foliating herbs | *Urtica laetevirens* | √ | √ | √ | √ |

**Appendix S3** Top 5 importance values of species under different nitrogen concentrations in the Korean pine plantation

| Spieces | Importance value (%) | | | | | | | | | |
| --- | --- | --- | --- | --- | --- | --- | --- | --- | --- | --- |
|  | 2015 | | | |  | 2021 | | | | |
|  | N0 | N20 | N40 | N80 |  | | N0 | N20 | N40 | N80 |
| *Adoxa moschatellina* | 16.32 | 14.01 | 16.11 | 6.42 |  | | — | 8.82 | 12.25 | 10.73 |
| *Aegopodium alpestre* | — | — | — | — |  | | 11.93 | — | — | 6.60 |
| *Anemone cathayensis* | — | — | — | — |  | | — | — | — | 9.12 |
| *Athyrium brevifrons* | 8.65 | — | 6.22 | 15.26 |  | | 12.56 | 18.51 | 13.79 | 8.98 |
| *Athyrium spinulosum* | — | 13.20 | 24.45 | — |  | | — | — | — | — |
| *Cardamine leucantha* | — | — | — | — |  | | 8.69 | — | — | — |
| *Chrysosplenium lectus-cochleae* | 7.42 | 7.21 | 7.53 | — |  | | 9.44 | 8.50 | 12.06 | — |
| *Enemion raddeanum* | — | — | — | 7.43 |  | | — | — | 6.61 | — |
| *Hylomecon japonica* | — | 6.59 | — | — |  | | — | — | — | — |
| *Mitella nuda* | 6.09 | — | — | — |  | | — | — | — | — |
| *Moehringia lateriflora* | — | — | — | — |  | | — | 6.64 | — | — |
| *Oxalis corniculata* | 13.54 | 11.58 | 6.83 | 6.57 |  | | 9.33 | 8.43 | 6.59 | — |
| *Pseudostellaria davidii* | — | — | — | — |  | | — | — | — | 6.99 |
| *Rubia sylvatica* | — | — | — | 6.29 |  | | — | — | — | — |
| Others | 41.31 | 47.41 | 32.20 | 44.70 |  | | 48.05 | 49.09 | 35.36 | 37.58 |

**Appendix S4** The linear mixed model results of the response of early spring herb species richness to nitrogen application rate and year under different life history strategies in 2015,2017,2019 and 2021. The t-values and p-values in parentheses are shown, bold values denote significant effects (*P* < 0.05).

| Effect | df |  | | Life-history strategy | | |
| --- | --- | --- | --- | --- | --- | --- |
|  |  | Early-spring growing herbs |  | Early-spring flowering herbs |  | Early-spring foliating herbs |
| Year | 76 | 3.820 (**<0.001**) |  | 3.996 (**<0.001**) |  | -2.631(**0.010**) |
| Nitrogen | 76 | 2.824 (**0.006**) |  | -0.673 (0.503) |  | 1.793 (0.077) |
| Nitrogen×Year | 76 | -3.535 (**<0.001**) |  | -1.789 (0.078) |  | 1.605 (0.113) |
